# Risk Factors of Ureteral Stenosis in Kidney Transplant Recipients: A Retrospective Study in National Referral Hospital in Indonesia

**DOI:** 10.1155/2021/2410951

**Published:** 2021-01-11

**Authors:** Gampo A. Irdam, Bobby Sutojo, Putu A. R. Raharja

**Affiliations:** Department of Urology, Faculty of Medicine Universitas Indonesia, Cipto Mangunkusumo Hospital, Jakarta 10430, Indonesia

## Abstract

Ureteral stenosis is one of the most common urological complications following kidney transplantations. It is occurred in 2–10% of patients and poses a significant problem to the patients as it may lead to permanent damage to renal damage. Identification of risk factors is important to prevent the incidence of ureteral stenosis. Thus, we aim to determine the risk factors of ureteral stenosis in the Indonesian population. This is a retrospective analysis of 487 kidney transplant patients performed in Cipto Mangunkusumo Hospital between 2014 and 2018. We collected and compared donor and recipient demography data in recipients who developed ureteral stenosis and recipients who did not develop ureteral stenosis. Ureteral stenosis was defined as the presence of hydronephrosis from ultrasound and increased number of serum creatinine. The overall incidence of ureteral stenosis post-kidney transplantation in our center is 6.6% (32 from 487 patients) from January 2014 until June 2018. We found that older donor and recipient age more frequent in developing ureteral stenosis post-kidney transplantation (*p* < 0.001). We also found that donors with number of arteries more than 2 (*p* < 0.001) and prolonged warm ischemic time (*p* < 0.05) are more frequently to develop ureteral stenosis post-kidney transplantation. There is no association between type II diabetes mellitus and hypertension with ureteral stenosis in this study. Donor age, recipient age, donor number of arteries more than 2, and prolonged warm ischemia time are associated with ureteral stenosis after kidney transplantation.

## 1. Introduction

End-stage renal disease (ESRD) is a worldwide public health problem with increasing prevalence each year. Based on the Medicare-funded program, there was an increase from approximately 10,000 cases in 1973 to 703,243 in 2015 in the United States [1]. In 2014, the Indonesian Renal Registry showed the annual ESRD incidence of 35,000 patients and prevalence of 120,000 patients [2]. There are some renal replacement therapy modalities for ESRD including hemodialysis, peritoneal dialysis, and kidney transplantation [1, 2]. Kidney transplantation has been considered as the best treatment for ESRD which provides not only better long-term survival but also a better quality of life than other types of renal replacement therapy [3, 4]. Acute graft rejection once becomes a major concern in kidney transplantation [5–7], but advances in the management of graft rejection have led to decreased number of graft failures and improvement of patient survival rates [5, 6]. Reduced number of graft failures apparently is followed by an increase in posttransplant urological complications [7]. The most common urological complication following kidney transplantation is ureteral stenosis [5], which occurred in 2–10% of patients receiving kidney transplantation [4]. Ureteral stenosis poses significant problems to patients with kidney transplantation as it may lead to permanent damage to the renal allograft [6].

Almost 70% of ureteral obstructions following kidney transplantation were observed within 3 months [8]. Ischemia is probably the main reason, but it is difficult to demonstrate this directly. Some authors reported donor age as the risk factor for stenosis [9], while some other authors denied this finding [10]. The number of allograft arteries is the other risk factor. Some authors even reported cases of ureteral stenosis caused by CMV infection [8]. However, no studies have been conducted in Indonesian population to identify the risk factors of ureteral stenosis after kidney transplantation. Thus, we aim to determine the risk factors of allograft ureteral stenosis following kidney transplantation in Indonesian population.

## 2. Methods

This is a retrospective cohort study of 487 renal transplants performed in Cipto Mangunkusumo Hospital (National Referral Hospital in Indonesia) between January 2014 and June 2018. The surgical transplantation technique was quite homogeneous during the entire study. We included all the patients that underwent kidney transplantation between the study time. Diagnosis of ureteral stenosis is defined as the presence of hydronephrosis (dilated renal calyces and pelvis) from ultrasound and increased number of serum creatinine and decreased urine output (<100 mL/day) [10]. These findings were then confirmed using antegrade or retrograde pyelography. All other causes of graft function impairment, i.e., acute rejection, hematoma, lymphocele, and urine collections, were ruled out. All patients were stented.

Transplantation procedure was done by the technique of anastomosis of the renal vein followed by renal artery anastomosis. We performed end-to-side anastomosis between the graft renal vein and the recipient's external iliac vein by extraperitoneal approach. The arterial anastomosis was done by end-to-side anastomosis to the external iliac artery. Extravesical ureteroneocystostomy was done by Lich–Gregoir technique. Vascular reconstruction in multiple renal arteries was done by side-to-side anastomosis on small vessels with end-to-side anastomosis of the smaller artery to the main artery, side-to-side anastomosis of similar-sized arteries, and anastomosis to the recipient internal, external, or common iliac and hypogastric arteries [11].

We collected the demographic data, intraoperative parameters, and postoperative parameters from both donor and recipient. Data were presented using tables. We then compared the demographic, intraoperative, and postoperative data with the occurrence of ureteral stenosis with statistical analysis using Statistical Package for the Social Sciences (SPSS) version 23. The data were compared, and the *p* value was measured to define the clinical significance. The data were presented in frequency and mean or median (mean if the data's distribution is normal and median if the distribution is abnormal). Continuous variables were compared using independent *t*-test if the data were normally distributed or Mann–Whitney test if the data were not normally distributed. Categorical variables were compared with chi-square test. *p* value less than 0.05 was considered statistically significant.

## 3. Results

The overall incidence of ureteral stenosis post-kidney transplantation in our center is 6.6% (32 from 487 patients) from January 2014 until June 2018. In this study, the median age of the donor is 31 (18–50) years; meanwhile, the median age of the recipient is 47 (8–78) years and majority of donor and recipient is male (69.6% and 73.3%, respectively). About 29.6% of the recipients had a history of diabetes mellitus and 60.8% had a history of hypertension. Only 5 recipients had neurogenic bladder condition. The demographic data of subjects are shown in Table 1.

The comparison of demographic data, intraoperative parameters, and postoperative parameters between ureteral stenosis and nonureteral stenosis groups is shown in Table 2. We found that there were significant differences in donor and recipient age in patients who develop ureteral stenosis following kidney transplantation. The older age of donor and recipient was more likely to develop ureteral stenosis postoperatively (*p* < 0.001). No difference was found in donor and recipient sex between the two groups (*p* = 0.2 and *p* = 0.85). The relationship between donor and recipient also did not play a significant role in developing ureteral stenosis after kidney transplantation (*p* = 0.68).

We found that donors with more than 2 allograft arteries were more frequently to develop ureteral stenosis post-kidney transplantation (12.5% vs. 0.9%, *p* < 0.001); meanwhile, the number of allograft veins did not play a significant role. We also found that prolonged warm ischemic time is correlated with the development of ureteral stenosis (*p* = 0.038). Recipient comorbidities (type 2 diabetes mellitus and hypertension) also did not seem to have a major role in causing ureteral stenosis postoperatively (*p* = 0.55 and *p* = 0.08, respectively). History of previous kidney transplantation also did not seem to increase the risk of ureteral stenosis (*p* = 0.47). The difference between preoperative and postoperative levels of creatinine in the two groups also did not show statistical significance (*p* = 0.65 and *p* = 0.94).

## 4. Discussion

In spite of recent advances in urological surgical technique, continuous improvement, and center's experience, ureteral stenosis remains the most common urological complication following kidney transplantation. We report an incidence of 6.8% (37 from 545 patients) of ureteral stenosis after kidney transplantation in our center from 2014 until 2018. This number is not much different from previously reported in the literature as Karam et al. and Fontana et al. reported incidence of ureteral stenosis in the first month after transplantation is between 2 and 7.5% [6, 7].

There are various etiologies of ureteral stenosis, as mentioned before, such as technical error and external compression (hematoma, lymphocele, abscess, kinking redundant ureter, ureteral stone, anastomotic edema, and ureteral ischemia) [12]. Ureteral ischemia is known as the most common etiology for ureteral stenosis, which accounted for 60–95% of cases of ureteral stenosis. The distal ureter is the most vulnerable part of the ureter to develop ureteral ischemia owing to its anatomical location that is the furthest part of the renal artery [5]. In our center, we also found many patients with impaired ureteral vascularization that might precipitate ureteral ischemia.

From this study, we found that donor age, recipient age, number of arteries > 2, and prolonged warm ischemia time were associated with ureteral stenosis after kidney transplantation. Arpalli et al [13] reported that advanced donor age has a higher risk for developing ureteral stenosis after kidney transplantation (Cox HR: 1.03 (1.01–1.05) *p* = 0.03). Fontana et al. also reported the same result that donor age >65 years is more likely to develop ureteral stenosis (*p* = 0.001). Advanced age donor has been established as the independent risk factor for ureteral obstruction following kidney transplantation from various literature studies. Older donors usually have vascular problems that favorably cause ureteral ischemia. Older donor kidneys are also usually more susceptible to damage from cold ischemia, and small differences can have a significant impact on organ function. It is also reported that there is a higher rate of lymphocele formation in older donor kidney transplant, possibly due to more difficult surgical resection in older patients. This is due to higher fragility of renal lymphatic vessels and more abundant perihilar fatty tissue [6, 7, 13].

Recipient donor age was also reported as an independent factor for developing ureteral stenosis. Arpalli reported that younger recipient age at donor is 3% more unlikely to develop a ureteral stenosis over 15 years (Cox HR: 0.97 (0.95–0.99) *p* = 0.01). This is due to more frequent vascular problems that can cause ureteral ischemia and due to underdiagnosed benign prostate hypertrophy (BPH) in previously anuric or oliguric patients that cause many posttransplantation complications [13].

More than two renal arteries were found to be associated with ureteral stenosis after kidney transplant in this study. Rahnemai-Azar et al. [14] also reported the same result (OR 2.48 (1.16–3.34), *p* = 0.02) that is explained by relative ischemia of the ureter due to malperfusion of accessory arteries from small anastomosis, greater turbulence, or more vulnerability of the arteries for traction injury. Multiple renal arteries, theoretically, pose a significant risk for postoperative risk after renal transplantation. Even at the beginning of the transplantation era, multiple renal arteries are considered as a contraindication. However, several techniques have been developed to reconstruct the multiple renal arteries and reported to significantly reduce vascular complications.

The clinical significance of these results can be taken for early diagnosis and prevention of ureteral stenosis after kidney transplantation in Indonesian population. Patients with high-risk profiles have to undergo several monitoring techniques to diagnose and evaluate the development of ureteral stenosis over time [15]. The modalities include ultrasonography, computed tomography scan, magnetic resonance urography, and scintigraphy. Identification of high-risk profiles can lead to more cost-efficient evaluation of patients with kidney transplantation. Also, prevention of ureteral stenosis can be taken by the placement of ureteral stent that has proven effective to prevent ureteral stenosis [16].

## 5. Conclusion

Donor age, recipient age, number of arteries > 2, and prolonged warm ischemia time are associated with ureteral stenosis after kidney transplantation.

## Figures and Tables

**Table 1:** Demographic data of the patients.

	Frequencies or mean
Donor age (years)	31 (18–50)
Recipient age (years)	47 (8–78)
Donor sex (male)	339 (69.6%)
Recipient sex (male)	357 (73.3%)
Recipient with type II diabetes mellitus	144 (29.6%)
Recipient with hypertension	396 (60.8%)
Recipient with neurogenic bladder	5 (1.03%)

**Table 2:** Comparison of demographic data, intraoperative parameters, and postoperative parameters between the ureteral stenosis group and nonureteral stenosis group.

	Ureteral stenosis (*n* = 32 patients)	Without ureteral stenosis (*n* = 455 patients)	*p* value
Donor age (years)	40.5 (24–50)	30 (18–50)	<0.001^*∗*^

Donor sex			
Male, *n* (%)	18 (56.3%)	321 (70.5%)	0.13
Female, *n* (%)	14 (43.7%)	134 (29.5%)	
Donor-recipient relationship			0.68
Not related, *n* (%)	6 (18. 8%)	99 (21.8%)	
Related, *n* (%)	26 (81. 2%)	356 (78.2%)	
Donor site of transplant			0.83
Right, *n* (%)	2 (6.3%)	33 (7.3%)	
Left, *n* (%)	30 (93.7%)	422 (92.7%)	
Donor history of abdominal surgery	2 (6.3%)	27 (5.9%)	0.94
Donor number of veins			0.40
>1, *n* (%)	1 (3.1%)	6 (1.3%)	
≤1, *n* (%)	31 (96.9%)	449 (98.7%)	
Donor number of arteries			<0.001^*∗*^
>2, *n* (%)	4 (12.5%)	4 (0.9%)	
≤2, *n* (%)	28 (87.5%)	451 (99.1%)	
Warm ischemic time (min)	2422 (1892–4473)	2295 (293–5523)	0.038^*∗*^
Cold ischemic time (min)	1737 (1658–1817)	1892 (836–6480)	0.42
Time to urinate (sec)	328 (151–506)	125 (32–982)	0.93
Donor body mass index (kg/m^2^)	19.9 (19–20.8)	23 (17.1–33.5)	0.62
Recipient preoperative creatinine (mg/dL)	6.1 (1–14.4)	5.35 (5.3–5.4)	0.65
Recipient postoperative creatinine (mg/dL)	1.25 (1.1–1.4)	1.1 (0.3–19)	0.94
Recipient length of prior dialysis (month)	52.5 (21–84)	10 (1–216)	0.75
Recipient resistive index	0.67 (0.61–0.73)	0.72 (0.06–0.91)	0.98
Recipient age (years)	44 (19–77)	48 (8–78)	<0.001^*∗*^
Recipient sex			0.85
Male, *n* (%)	23 (71.9%)	334 (73.4%)	
Female, *n* (%)	9 (28.1%)	121 (26.6%)	
Recipient history of type II DM			0.55
Yes, *n* (%)	8 (25%)	136 (29.9%)	
No, *n* (%)	24 (75%)	319 (70.1%)	
Recipient history of HT			0.08
Yes, *n* (%)	24 (75%)	272 (59.8%)	
No, *n* (%)	8 (25%)	183 (40.2%)	
Recipient history of transplantation			0.47
Yes, *n* (%)	2 (6.3%)	17 (3.7%)	
No, *n* (%)	30 (93.7%)	438 (96.3%)	

^*∗*^Significant difference.

## Data Availability

The data used to support the findings of this study are available from the corresponding author upon request.
